# Genome-wide SNP data of Izumo and Makurazaki populations support inner-dual structure model for origin of Yamato people

**DOI:** 10.1038/s10038-020-00898-3

**Published:** 2021-01-25

**Authors:** Timothy Jinam, Yosuke Kawai, Yoichiro Kamatani, Shunro Sonoda, Kanro Makisumi, Hideya Sameshima, Katsushi Tokunaga, Naruya Saitou

**Affiliations:** 1 Population Genetics Laboratory, National Institute of Genetics, Yata 1111, Mishima, 411-8540 Japan; 2 Department of Genetics, School of Life Science, Graduate University for Advanced Studies (SOKENDAI), Yata 1111, Mishima, 411-8540 Japan; 3 Genome Medical Science Project, National Center for Global Health and Medicine, 1-21-1 Toyama, Shinjuku-ku, Tokyo, 162-8655 Japan; 4 Department of Computational Biology and Medical Sciences, Graduate School of Frontier Sciences, The University of Tokyo, 4-6-1 Shirokane-dai, Minato-ku, 108-8639 Japan; 5 Laboratory for Statistical and Translational Genetics, RIKEN Center for Integrative Medical Sciences, 1-7-22 Suehiro-cho, Tsurumi-ku, Yokohama, 230-0045 Japan; 6 The Makurazaki City Medical Association, 102-1, Kotobuki-cho, Makurazaki-shi, 898-0062 Japan; 7 Faculty of Medicine, University of the Ryukyus, 207 Uehara, Nishihara-cho, 903-0215 Japan; 8 Department of Biological Sciences, Graduate School of Science, the University of Tokyo, 7-3-1 Hongo, Bunkyo-ku, Tokyo, 113-8655 Japan

**Keywords:** Evolutionary biology, Genome informatics

## Abstract

The “Dual Structure” model on the formation of the modern Japanese population assumes that the indigenous hunter-gathering population (symbolized as Jomon people) admixed with rice-farming population (symbolized as Yayoi people) who migrated from the Asian continent after the Yayoi period started. The Jomon component remained high both in Ainu and Okinawa people who mainly reside in northern and southern Japan, respectively, while the Yayoi component is higher in the mainland Japanese (Yamato people). The model has been well supported by genetic data, but the Yamato population was mostly represented by people from Tokyo area. We generated new genome-wide SNP data using Japonica Array for 45 individuals in Izumo City of Shimane Prefecture and for 72 individuals in Makurazaki City of Kagoshima Prefecture in Southern Kyushu, and compared these data with those of other human populations in East Asia, including BioBank Japan data. Using principal component analysis, phylogenetic network, and f4 tests, we found that Izumo, Makurazaki, and Tohoku populations are slightly differentiated from Kanto (including Tokyo), Tokai, and Kinki regions. These results suggest the substructure within Mainland Japanese maybe caused by multiple migration events from the Asian continent following the Jomon period, and we propose a modified version of “Dual Structure” model called the “Inner-Dual Structure” model.

## Introduction

**Fig. 1:**
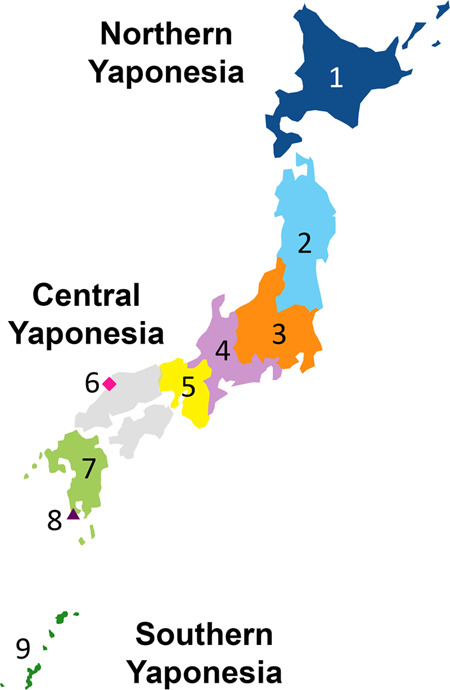
Geographical map of Yaponesia. The different regions are color-coded and numbered to correspond to Fig. 3

The Japanese Archipelago spans more than 2000 km from north to south. This Archipelago was called “Yaponesia” by writer Toshio Shimao in early 1960s [1], by connecting “Yapo” (Japan in Latin) and “nesia” (islands in Latin). Yaponesia can be divided into three geographical areas: Northern Yaponesia, Central Yaponesia, and Southern Yaponesia (Fig. 1). Northern Yaponesia consists of Hokkaido (sometimes Sakhalin and Kuril Islands may also be included [2]), Central Yaponesia consists of Honshu, Shikoku, and Kyushu, and Southern Yaponesia consists of the islands in Okinawa prefecture, also known as Ryukyu Islands. Besides people from Okinawa and the Ainu from Hokkaido, people who inhabit the Japanese Archipelago can be referred to as Yamato people.

**Fig. 2:**
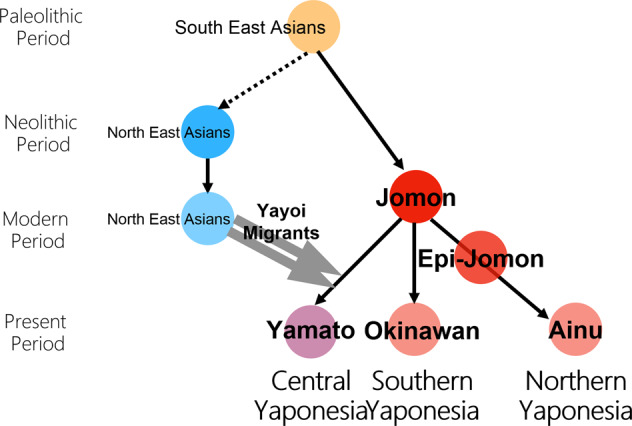
Re-drawing of the dual structure model from Hanihara [5]

Regarding the formation of Yaponesians (Japanese), von Baelz [3] first pointed out common features in Ainu and Okinawan people as early as 1911. This view was later supported by many researchers, and renamed by Yamaguchi [4] and Hanihara [5] as the “Dual Structure” model. This model has often been invoked to explain the genetic diversity of people from the Japanese Archipelago, which has been inhabited by humans long before the Jomon period (16,000–3,000 years before present, BP [6, 7]). According to the model, rice agriculturists (symbolically called “Yayoi” people) migrated from the Asian Continent during the Yayoi period (10th century B.C.–3rd century A.D. [7]). They admixed with the indigenous hunter-gatherers (symbolically called “Jomon” people), resulting in the current Japanese population. Figure 2 shows the “Dual Structure” model based on Hanihara [5]. This model also posits that Ainu people from Hokkaido in the north and Okinawa people from the southernmost islands have the much higher proportion of Jomon ancestry compared to Yamato people. Initially conceived from craniometric data, the model now has a strong support from our genome-wide SNP data [8].

However, most genetic data of Yamato people were from Tokyo and its surrounding Kanto region. There have been reports of substructure within Japanese people using genome-wide SNPs [9, 10] and HLA alleles [11, 12]. Nonetheless, some regions in Japan are under-represented in population genetics studies. We therefore generated new genome-wide SNP data from people in Izumo, Shimane Prefecture and Makurazaki, Kagoshima Prefecture, and together with data from other parts of Japan, we tested whether the “Dual Structure” model still applies to these populations.

## Materials and methods

A total of 90 and 72 individuals from Izumo City of Shimane Prefecture and Makurazaki City of Kagoshima Prefecture, respectively (see Fig. 1), were recruited for this study.

It should be noted that individuals from Izumo were sampled in two periods and at different locations. We first collected blood samples at Department of Human Genetics, School of Medicine, the University of Tokyo in 2013 from 21 individuals who grew up in Izumo City and were at that time residents of Tokyo Area. Most of their grandparents also grew up in Izumo area. These data on 21 individuals were reported by Saitou and Jinam [13] as a preliminary study. We later collected saliva samples at the Archeological Museum of Kojindani, Izumo City in 2014 from 69 individuals. In total, only 51 DNA samples from Izumo contained enough good-quality DNA for genotyping.

As for the Makurazaki population, blood samples were collected from 72 individuals in 2015 at Makurazaki City with the help of the Makurazaki City Medical Association, and all 72 individual DNA samples were used for this study. Four grandparents of all these 72 individuals collected in Makurazaki grew up in the Southern Satsuma region. Both the Izumo and Makurazaki samples were genotyped using the Japonica array [14]. After filtering out SNPs based on genotyping call rate (<95%) and the Hardy-Weinberg equilibrium (*p* < 1 × 10^−10^), a total of 625,177 autosomal SNPs remained. Cryptic relatedness within each population was checked using KING software [15]. Six individuals from Izumo and eight from Makurazaki with up to 3rd degree relations (kinship coefficient more than 0.06) were omitted from further analysis. Therefore, 45 and 64 genome-wide SNP data for Izumo and Makurazaki, respectively, were used for subsequent data analyzes.

The Japonica-array SNP data for Izumo and Makurazaki were then merged with the following datasets: (1) BioBank Japan (BBJ) [16, 17] genome-wide SNP data (922,511 sites); (2) Affymetrix 6.0 micro-array data (641,314 SNP sites) of 20 Ainu, 35 Okinawa, 50 Yamato (mostly from Kanto region) [8], and 42 Chinese Han in Beijing (CHB) [18] individuals; (3) high-coverage whole genome data of 88 Koreans from the Korean Personal Genome Project [19]; (4) high-coverage whole genome data of Asian populations (CHB, Southern Han Chinese, Chinese Dai from China, Kinh from Vietnam) from the 1000 genomes project [20].

The BioBank Japan dataset (BBJ) [17] consisted of 182,546 individuals treated at hospitals in seven regions in Japan: Hokkaido, Tohoku, Kanto-Koshinetsu (Kanto for short), Tokai-Hokuriku (Tokai for short), Kinki, Kyushu, and Okinawa (Fig. 1). The genotyping data obtained by SNP array was downloaded from Japanese genotype-phenotype archive on the approval of NBDC Data Access Committee (JGA Accession ID: JGAD000123). The genotype data were converted into VCF format.

Whole genome sequence data were analyzed by the workflow recommended by the GATK best practice. We retrieved the mapped-read data in CRAM format for Asian population from the 1000 Genomes project (https://www.internationalgenome.org/). For Korean population, raw reads in fastq format were retrieved from The Personal Genome Project Korea (http://opengenome.net/Main_Page). The raw reads were then mapped to GRCh38 reference sequence by bwa-mem in order to consolidate with 1000 Genomes project data. Variant discovery was performed using the HaplotypeCaller algorithm implemented in GATK4.1 to perform joint calling. Joint calling was performed by GVCFtyper algorithm implemented in sentieon package [21] for computational efficiency. This program yields results compatible with GATK’s GenomicsDB and genotypeGVCFs programs. Each variant was scored using the VQSR algorithm to filter the variants. We applied VQSR by valCal and ApplyVarCal program of sentieon package. The HapMap and International 1000 Genomes Omni2.5 sites, the high-confidence SNPs of 1000-G sites, and the dbSNP151 sites are used as true datasets, training datasets, and known datasets, respectively. SNPs that passed the 99.9% sensitivity filter by VQSR were used for subsequent analyzes. The filtered genotype data in VCF were then merged with SNP array data of the BBJ, Izumo, and Makurazaki datasets by the merge function implemented in bcftools. SNPs that are missing in either dataset, minor allele frequency <1%, call rate <3%, or statistically significant deviation from Hardy-Weinberg equilibrium (*p* value < 0.00001) were filtered out. In total 106,237 SNPs remained. For the subsequent analyzes that require the independence of nearby SNPs, the linkage disequilibrium (LD)-based SNP pruning were performed by PLINK1.9 [22] with “--indep-pairwise 500 50 0.1”. We obtained 39,331 LD independent SNPs. After excluding duplicate samples and those with cryptic relatedness by KING, the principal component analysis (PCA) was performed using PLINK1.9 to remove outlier samples. The number of BBJ samples remaining after this step was 169,169. To reduce computation times for subsequent analyzes, we decided to further trim down the BBJ dataset. First, the mean values of the top 10 principal component values were calculated for each region, and the deviation of the principal component values from the mean were assessed for each sample by Euclidean distance. The top 1000 samples with the smallest Euclidean distances were selected from each region and used for subsequent analyses.

PCA was again performed using the trimmed down BBJ dataset using PLINK1.9, and Admixture [23] was used for admixture analysis. f4 test implemented in treemix [24] was used to test for gene flow between populations. GBR (British in England and Scotland) population from the 1000 Genomes dataset was used as outgroup for the f4 test. Phylogenetic analysis of populations was conducted using the neighbor-joining method [25] and Neighbor-Net [26] for phylogenetic tree and network construction, respectively. Pairwise Fst distances between populations calculated in smartpca [27] was used for tree and network construction.

## Results

**Fig. 3:**
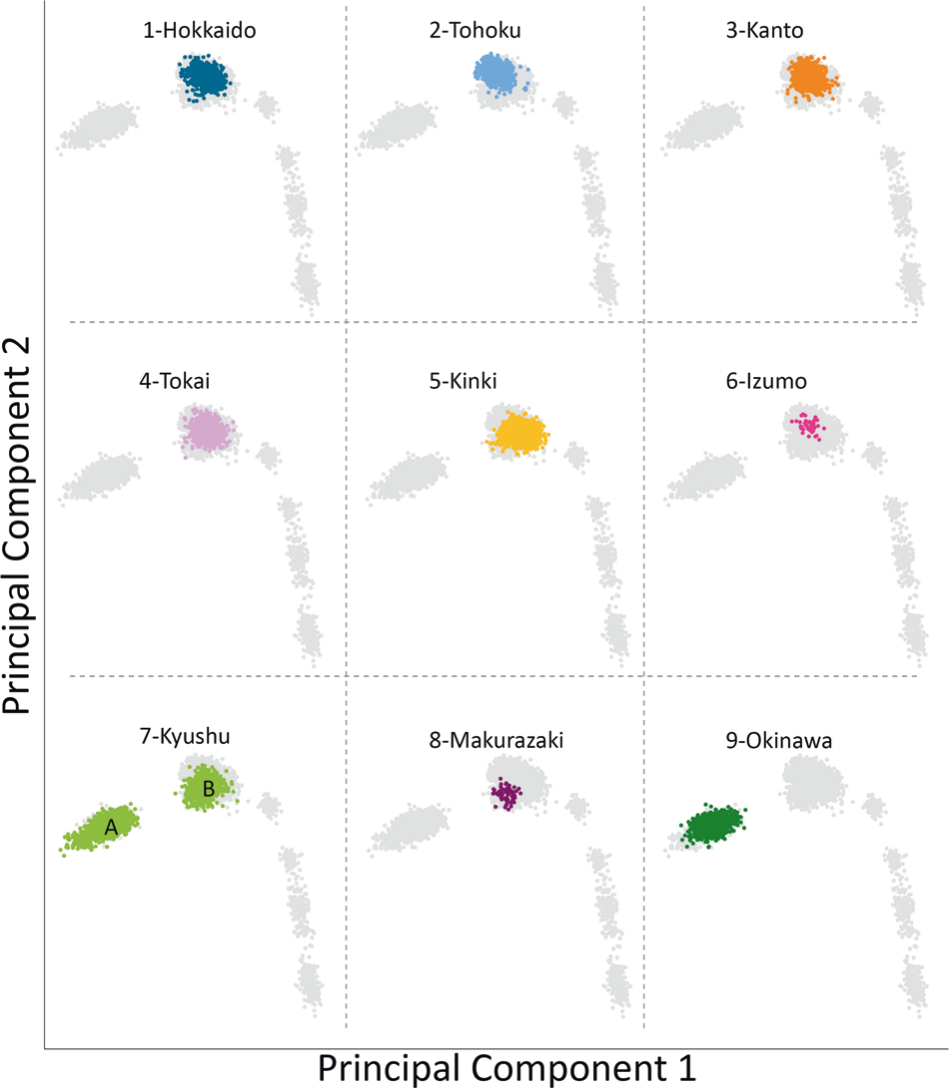
Principal component analysis (PCA) plots of individuals from various regions in Japan corresponding to Fig. 1 map. Non-Japanese individuals are indicated by gray dots. Two major clusters were observed in the BioBank Japan Kyushu cohort, labeled A and B, respectively

We first conducted PCA under various combinations of populations. Supplementary Fig. 1 shows that Makurazaki and Izumo cluster with Yamato when PCA was performed with Ainu, Okinawan, and CHB. Next, PCA was performed using Izumo, Makurazaki, BBJ, Korean, and 1,000 genomes project data (Fig. 3). A total of 106,237 SNP sites were used for this PCA. Non-Japanese individuals are represented by gray dots and the detailed positions are shown in Supplementary Fig. 2, while the nine Yaponesian populations (see Fig. 1 for their geographical locations) are shown as different colors in each panel. The left most cluster in each panel is made up of Okinawa (9) individuals, Yamato individuals in the middle cluster, while other continental Asians are located on the right side. Individuals from Hokkaido (1), Kanto (3), and Tokai (4) are distributed in the center of Yamato cluster, while Tohoku (2) and Kinki (5) individuals are slightly deviated from the central cluster. Interestingly, individuals sampled from Kyushu (7) are located both in Okinawa and Yamato clusters (shown as A and B, respectively). These Kyushu individuals who are within the Okinawa cluster (referred to as “Kyushu-A” hereafter) might have been sampled from islands that are geographically close to the Okinawa but are included administratively in Kagoshima prefecture of Kyushu. For BBJ data, geographical location of samples reflect the locations of hospitals where the patients were recruited from. In contrast, we collected DNA from Izumo and Makurazaki individuals only if all four grandparents were residents of their respective regions, a similar strategy employed in a study of British populations [28]. Makurazaki is located at the southern tip of Kyushu (see Fig. 1) and Fig. 3 shows that individuals of this city are located slightly nearer to the Okinawa cluster.

**Fig. 4:**
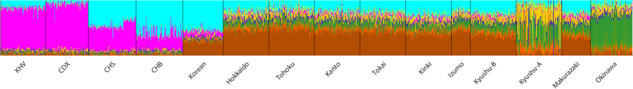
Admixture analysis for k (number of ancestral components) = 9. For clarity, only 100 randomly selected individuals from each region of the BioBank Japan dataset are included in this figure

The results of admixture analysis [23] when k (number of ancestral components) equals to nine is shown in Fig. 4. Cross validation errors for other values k are shown in Supplementary Fig. 3. The main ancestry components in Yaponesians (more than 10%) are indicated in brown, light blue and dark green colored ancestry components. The light blue ancestry component is highest in northern East Asians (CHB and Koreans) and on average 17% in Yaponesians except for individuals belonging to Okinawa and Kyushu-A. Another ancestry component (brown) is present at 42% in Yamato and 25% in Koreans but very low in Okinawa, Kyushu-A, and other Asian populations. The dark green ancestry component is highest in Okinawans, and Yamato populations consistently have this component, whereas the yellow ancestry component is highest among Kyushu-A population.

**Fig. 5:**
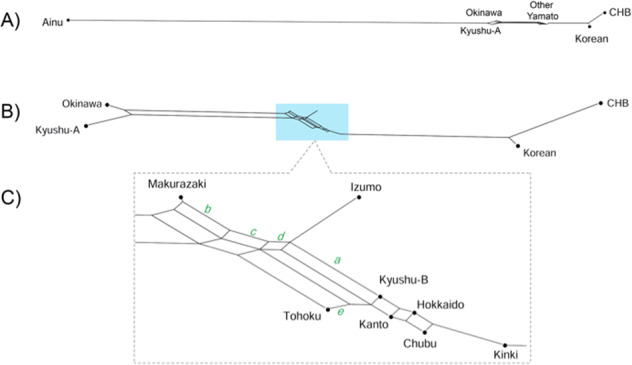
Neighbor-Net network from pairwise Fst distances between populations. **A** Network when all populations were included; **B** Network after Ainu was omitted; **C** Magnified view of reticulations involving Yamato people

An unrooted tree was constructed using the neighbor-joining method [25] based on Fst distances between populations (Supplementary Fig. 4). Populations are located more or less in a linear fashion; Okinawa/Kyushu-A cluster on the left and the CHB/Korean cluster at the right. Yamato people are in the center, with Kinki being the closest to the CHB/Korean cluster. We also constructed phylogenetic networks using Neighbor-Net method [26] focusing on Yaponesians and Korean/CHB (Fig. 5) and their relationship with other continental Asians (Supplementary Fig. 5). Because the Ainu population is very distant from the remaining populations (Fig. 5A), they were omitted from the subsequent network (Fig. 5B). Now Okinawa and Kyushu-A populations formed one cluster, and the remaining seven Yaponesian populations (shown in light blue background in Fig. 5B) are located between this cluster and the Korean-CHB cluster. Figure 5c shows the phylogenetic relationship of these seven Yaponesian populations. It should be noted that BBJ data for Fig. 5 were sampled in a different way than that for the remaining figures; 100 individuals were randomly chosen from seven populations of BioBank. This sampling difference probably caused the change of the Tohoku population; either somewhat closer to Izumo in Fig. 5C or almost identical to JPT in Supplementary Fig. 5. Makurazaki is the closest to the Okinawa and Kyushu-A cluster (indicated by splits labeled *b*, *c*, and *d*), followed by Izumo as shown by split *a*. Okinawa, Kyushu-A, and Tohoku are separated from the remaining nine populations by split *e*.

The results of f4 test for gene flow are listed in Table 1. To test if there is gene flow between Yaponesians and continental Asians, we tested f4(GBR, CHB; Okinawa, x) where x is the test population. Consistent with PCA, phylogenetic trees and network, Kinki showed the highest Z-score (26.2) among the Yamato populations. Similarly, f4(GBR,CHB;x1,x2) where x1 and x2 are non-Okinawan Yaponesians also showed evidence of gene flow between Kinki and CHB (Supplementary Table). For example, most significant Z*-*scores were observed for f4(GBR,CHB; Kanto, Kinki), Z-score: 24.7. We also tested f4(GBR, x; Izumo, Kinki) to test if there is gene flow involving Izumo. Negative f4 values indicative of gene flow was observed for Okinawa, Kyushu-A, and Makurazaki, although the Z-scores are relatively low. This was repeated with Makurazaki instead of Izumo using f4(GBR, x; Kinki, Makurazaki), and results suggest presence of gene flow between Makurazaki and Okinawa or Kyushu-A. Gene flow between Okinawa and Kyushu-A was further supported by f4(GBR, Okinawa; x1, x2) where x1 and x2 are non-Okinawa Yaponesians (Supplmenentary Table).

**Table 1:** f4 test for gene flow

*x*	f4	Std. error	*Z*-score
f4(GBR, CHB; Okinawa, x)			
Hokkaido	0.00124	5.07E−05	24.49
Tohoku	0.00111	4.79E−05	23.16
Kanto	0.00124	5.06E−05	24.48
Tokai	0.00127	5.17E−05	24.58
Kinki	0.00143	5.44E−05	26.20*
Izumo	0.00100	7.12E−05	14.08
Kyushu-A	0.00003	2.16E−05	1.16
Kyushu-B	0.00124	4.94E−05	25.12
Makurazaki	0.00111	5.82E−05	19.05
f4(GBR, x; Izumo, Kinki)			
Hokkaido	0.00008	4.89E−05	1.58
Tohoku	0.00005	4.92E−05	0.97
Kanto	0.00007	4.90E−05	1.44
Tokai	0.00009	4.89E−05	1.74
Kyushu-A	−0.00008	5.14E−05	−1.63
Kyushu-B	0.00009	4.89E−05	1.78
Makurazaki	−0.00003	5.00E−05	−0.69
Okinawa	−0.00008	5.12E−05	−1.64*
f4(GBR, x; Kinki, Makurazaki)			
Hokkaido	−0.00015	4.28E−05	−3.49
Tohoku	−0.00013	4.29E−05	−3.03
Kanto	−0.00014	4.28E−05	−3.33
Tokai	−0.00015	4.28E−05	−3.46
Izumo	−0.00002	4.50E−05	−0.41
Kyushu-A	0.00020	4.44E−05	4.45
Kyushu-B	−0.00010	4.27E−05	−2.35
Okinawa	0.00022	4.38E−05	4.92*

*Most significant Z-scores

## Discussion

We generated genome-wide SNP data for Izumo in the San-in region and Makurazaki in the southern Kyushu region (see Fig. 1) using Japonica array. The genetic location of Makurazaki population in the PCA plot (Fig. 3) and phylogenetic network (Fig. 5) is more or less similar to the geographical location of this city, namely, between Okinawa and the mainland. This is supported by f4(GBR, x; Kinki, Makurazaki) test showing Makurazaki having more gene flow with Okinawa and Kyushu-A than with other Yamato.

Geographically, Izumo city is facing the sea between Yaponesia and Continental East Asia (Fig. 1). The distance to the Korean Peninsula is ~350 km, and it was expected that Izumo people may be closer to continental Asians. However, f4 test showed that the Kinki population is the closest to continental Asians (f4(GBR, CHB; Okinawa, x)). This is supported by another study using genome-wide SNPs [29]. Interestingly, Izumo population has higher genetic affinity to Okinawa and Kyushu than to other populations in Honshu, as suggested by f4(GBR, x; Izumo, Kinki). This discrepancy suggests an existence of some gene flow events that are different from usually expected isolation by distance [30].

Yamaguchi-Kabata et al. [9] analyzed genome-wide SNP data of 7,000 Japanese collected from seven geographical regions (Hokkaido, Tohoku, Kanto, Tokai, Kinki, Kyushu, and Okinawa) as part of the BBJ Project. They found the two clear clusters called Hondo and Ryukyu, which we also observed (Fig. 3). We also confirmed region specific characteristics discovered by Yamaguchi-Kabata et al. [9] and later confirmed by Okada et al. [31] in which Hokkaido, Kanto, and Tokai area individuals are more or less genetically similar, whereas Tohoku individuals are slightly differentiated from those three regions people based on PCA, network and f4 tests. We also confirm that Kinki people are the closest to continental Asians as reported by Yamaguchi-Kabata et al. [9] and Watanabe et al. [29]. Kinki area includes Nara and Kyoto, past capitals of Japan and thus has a history of human migration from the Asian continent.

**Fig. 6:**
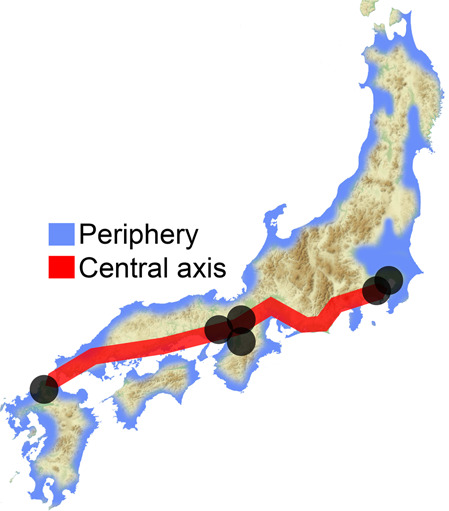
Representation of “Inner-Dual Structure” model

These results and HLA data [11] prompted Saitou [32] to propose the three-migration model. Later, Saitou and Jinam [13] and Saitou [2] proposed the “Inner-Dual Structure” model of Yaponesians, shown in Fig. 6. After the initial peopling of the Japanese Archipelago by the ancestors of Jomon and possibly including the paleolithic period people (first migration), there were possibly two (or more) human migrations from the Asian continent. Although the timing of this event is not yet determined, these people may have occupied coastal areas of the main island Honshu (“Periphery” in Fig. 6). Then the third migration from the continent mostly involved movement between the Asian Continent and six areas (Hakata in northern Kyushu, Osaka, Kyoto, Nara in Kinki, Kamakura, and Edo-Tokyo in Kanto; shown in gray circles in Fig. 6). These “Central Axis” areas were historically the cultural and political centers in Yaponesia, and are assumed to attract many new migrants from continental East Asia.

Two additional data also supported the “Inner-Dual Structure” model according to Saitou [2]; five mtDNA haplogroup frequency data of 18,641 Yaponesians sampled from all the 47 prefectures provided by Genesis Healthcare (see Supplementary Fig. 6; taken from Fig 52 of Saitou [2]) and ABO blood group allele frequency data of 46 prefectures except for Okinawa by Fujita et al. [33] and those for Okinawa Prefecture [34, 35] (see Supplementary Fig. 7; taken from Figure 53 of Saitou [2]). We divided the 47 prefectures into 26 peripheral (shown in blue) and 21 central axis (shown in red) in both data. In Supplementary Fig. 6, Principal Component 1 broadly separates peripheral (right side) and central axis (left side) populations. This bias is statistically significant at the 1% level using Fisher’s exact test (see the upper right 2 × 2 table of Supplementary Fig. 6). The frequency plot of alleles A and B of the ABO blood group (Supplementary Fig. 7) tend to group peripheral prefectures (shown in blue) to the lower left of the diagonal dotted line, and this bias is also statistically significant at the 1% level using Fisher’s exact test (see the upper right 2 × 2 table of Supplementary Fig. 7).

Recently, HLA data for all 47 prefectures were analyzed [12], and a phylogenetic relationship of 15 regions was shown. Interestingly, populations in northern Kyushu and southern Kanto are phylogenetically closer to continental Asian populations, whereas northern Tohoku, Shikoku and Okinawa formed one cluster. These patterns are compatible with the “Inner-Dual Structure” model shown in Fig. 6. However, San-in (belonging to Periphery in Fig. 6) and San-yo (belonging to Central Axis in Fig. 6) populations in Chugoku region are clustered in the phylogenetic tree of Hashimito et al. [12].

The “Inner-Dual Structure” model might explain why people in the Central Axis region appear closely related in the PCA and network analyzes, whereas people from the Periphery such as Makurazaki, Izumo, or Tohoku were less genetically influenced by the more recent migrations. However it is still not clear when those new and old migrants came to Yaponesia. Overall, these results appear to support the “Inner-Dual Structure” model, however other explanations such as very recent migrations might contribute to the observed patterns. The addition of samples from under-represented regions in Japan together with whole genome sequence data is needed to properly decipher the genetic make-up of Yaponesians.

## Supplementary information

Supplementary Tables

Supplementary Figures
