# Supplementary material for: Genome-wide SNP data of Izumo and Makurazaki populations support inner-dual structure model for origin of Yamato people
Source: J Hum Genet. 2021 Jan 25;66(7):681–7. doi: 10.1038/s10038-020-00898-3 (PMC8225512; doi:10.1038/s10038-020-00898-3)
Supplement: Supplementary file 2 — Supplementary Figures [file 10038_2020_898_MOESM2_ESM.pdf]

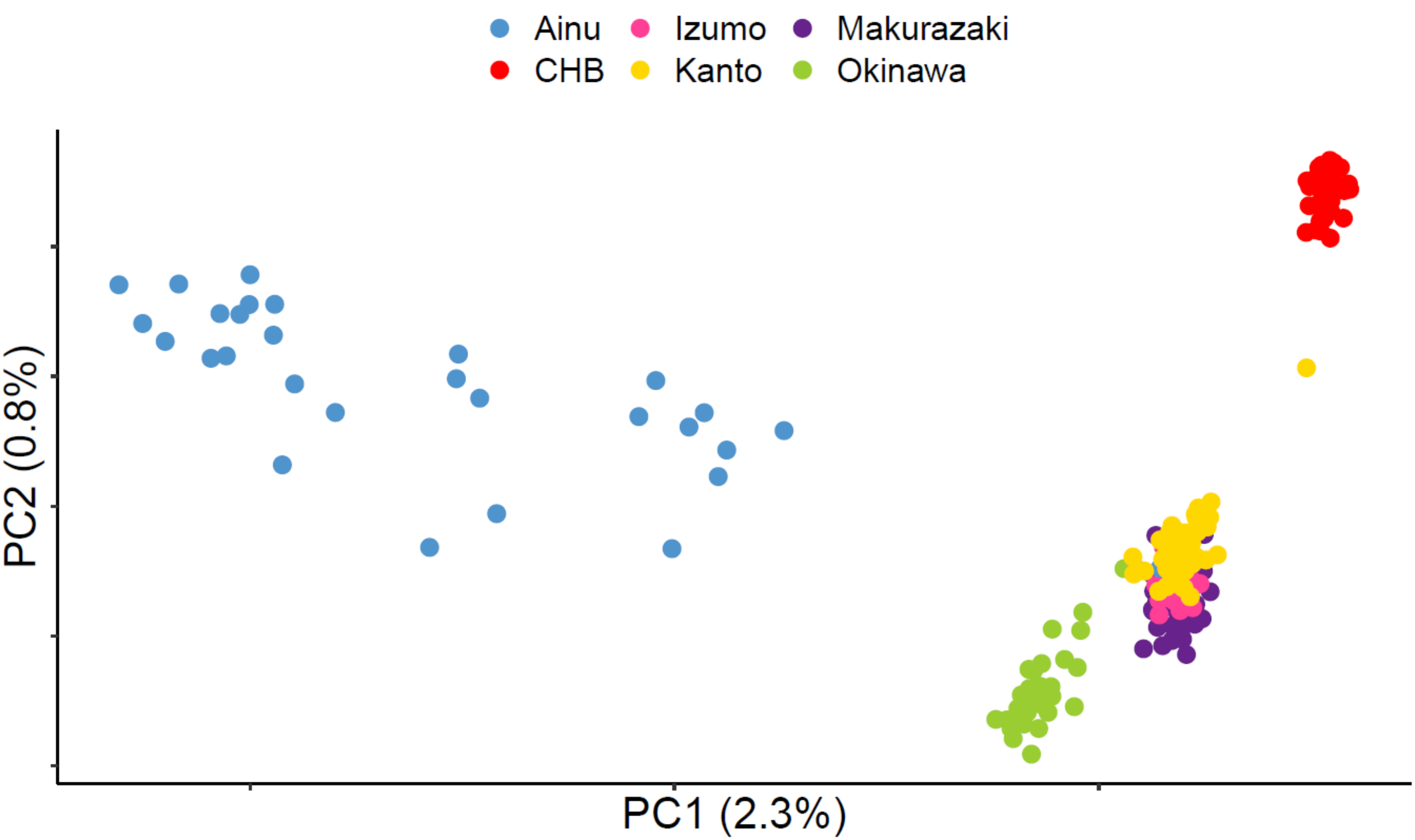

Supplementary figure 1: PCA plot of Ainu, Okinawa, Kanto, Makurazaki, Izumo and CHB

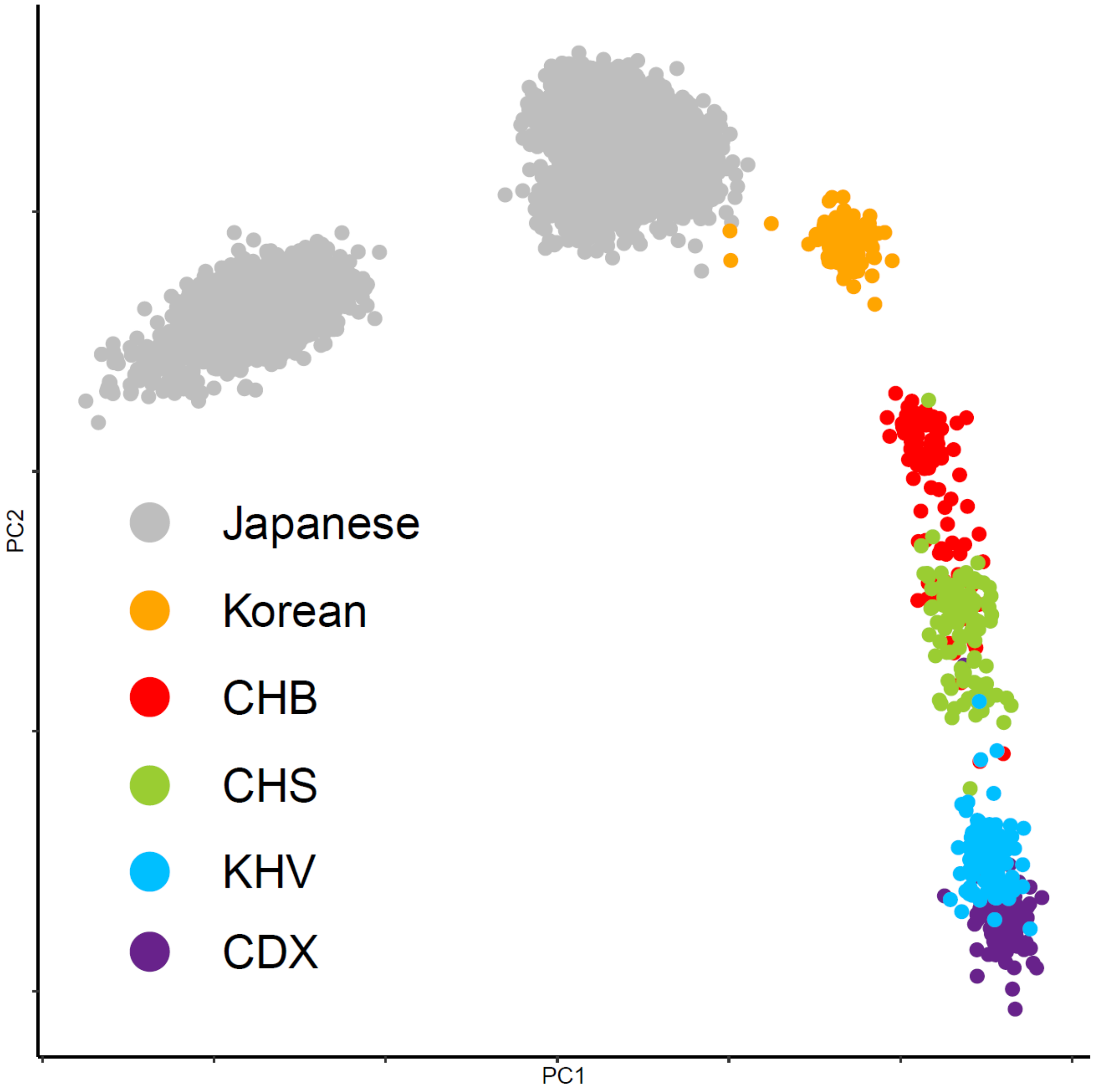

Supplementary figure 2: Details of non-Japanese populations from Fig. 3.

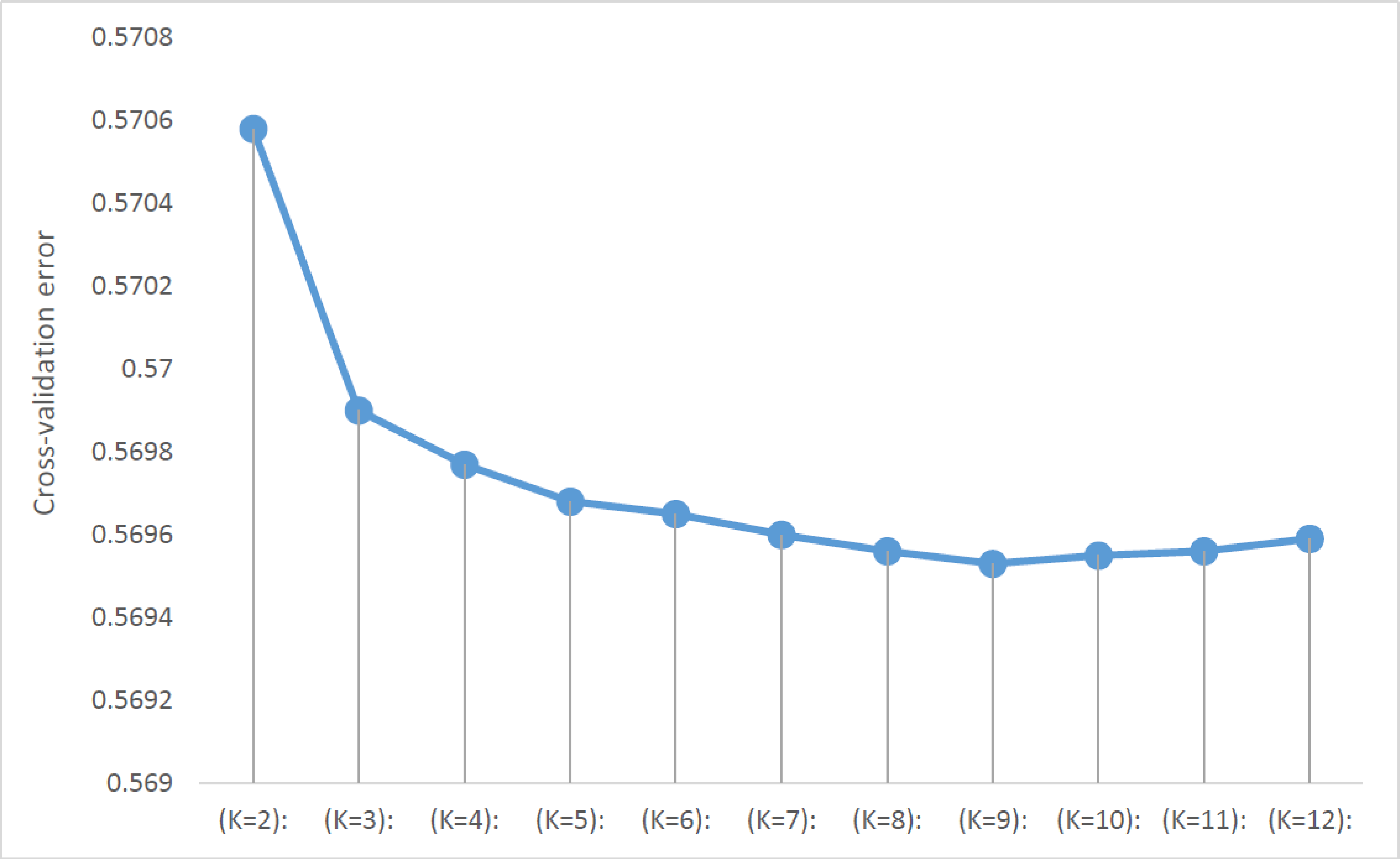

Supplementary figure 3: Cross validation error for Admixture runs assuming k=2 to k=12

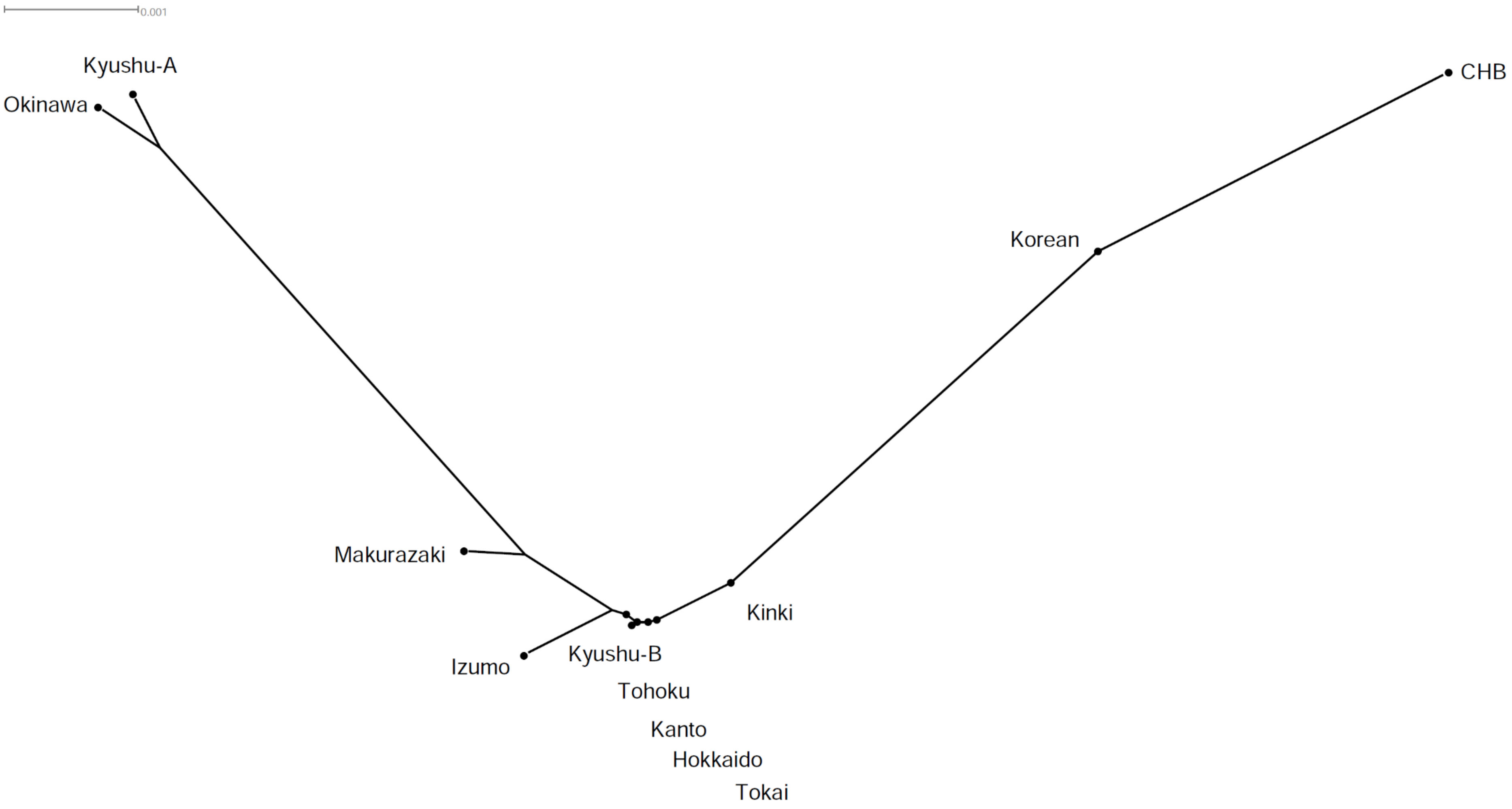

Supplementary figure 4: Neighbor-joining tree from Fst distances between populations

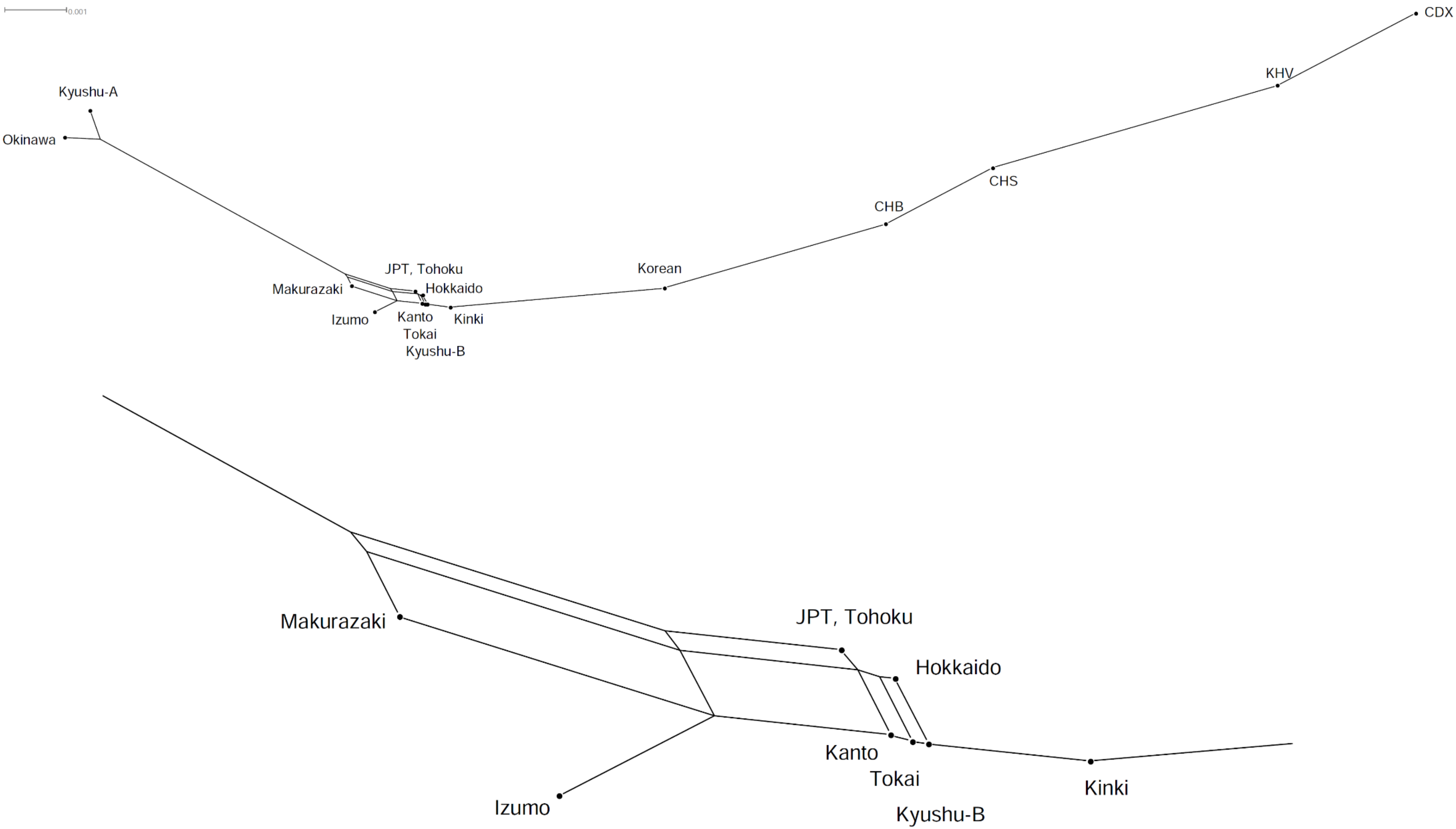

Supplementary figure 5: Neighbor-net networks from Fst distances between populations. Bottom panel shows magnified view of the central network involving Yamato populations.

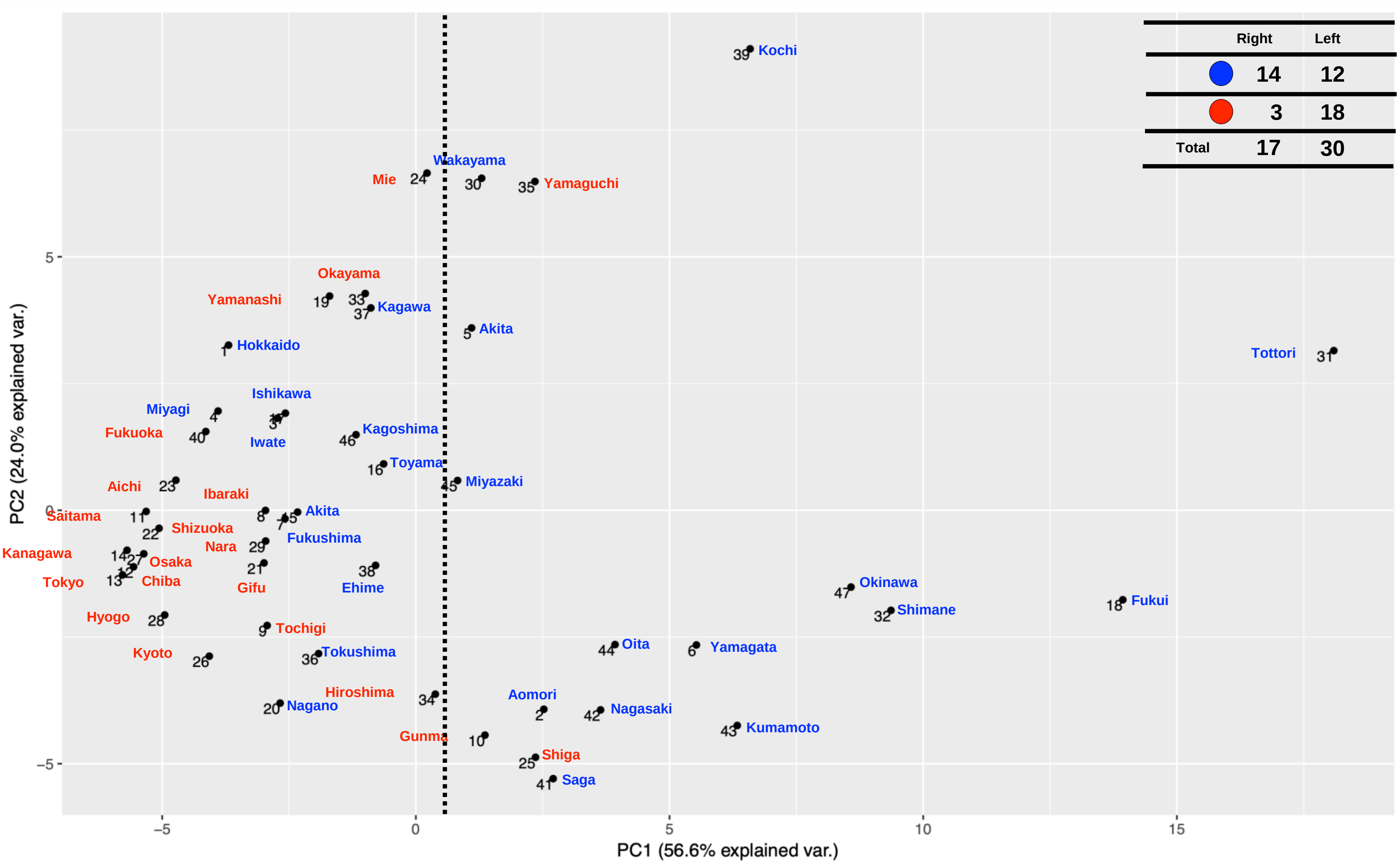

Supplementary Figure 6: PCA plot based on mtDNA haplotype frequencies of 18,641 Yaponesians (from Fig. 52 of Saitou [2017]). Red and blue texts correspond to “Central axis” and “Periphery” prefectures, respectively. These 47 dots were divided into right and left groups according to the vertical dotted line, and Fisher’s exact test for 2 x 2 table (shown in the upper right) showed statistical significance at the 1% level.

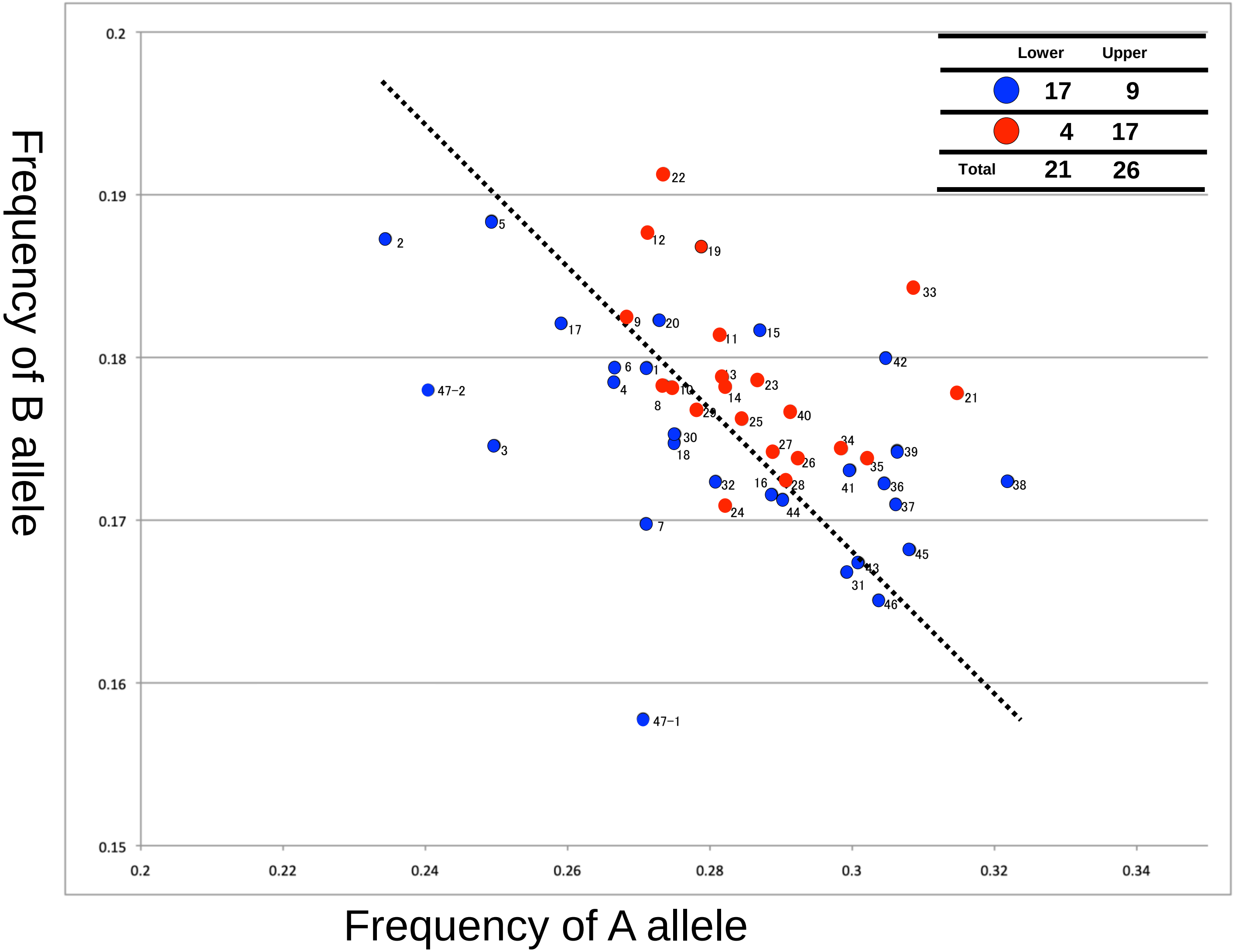

Supplementary Figure 7: Frequency plot of ABO blood group alleles A and B of 4.46 million Yaponesians (from Fig. 53 of Saitou [2017] with some modifications). Prefecture numbering 1-47 are the same as those shown in Supplementary Figure 6. There are two data for Okinawa Prefecture, shown as 47-1 and 47-2 (see text for detail). Red and blue texts correspond to “Central axis” and “Periphery” prefectures, respectively. These 47 dots (Okinawa was considered as one dot) were divided into upper and lower groups according to the diagonal dotted line, and Fisher’s exact test for 2 x 2 table (shown in the upper right) showed statistical significance at the 1% level.
